# Novel likely disease-causing *CLN5* variants identified in Pakistani patients with neuronal ceroid lipofuscinosis

**DOI:** 10.1016/j.jns.2020.116826

**Published:** 2020-07-15

**Authors:** Beenish Azad, Stephanie Efthymiou, Tipu Sultan, Marcello Scala, Javeria Raza Alvi, Caroline Neuray, Natalia Dominik, SYNaPS Study Group, Asma Gul, Henry Houlden

**Affiliations:** a Department of Biological Sciences, International Islamic University Islamabad, H-10, Islamabad 44000, Pakistan; b Department of Neuromuscular disorders, UCL Institute of Neurology, Queen Square, London WC1N 3BG, UK; c Department of Pediatric Neurology, The Children's Hospital and Institute of Child Health, Lahore 54600, Pakistan; d Pediatric Neurology and Muscular Diseases Unit, IRCCS Istituto Giannina Gaslini, Genoa, Italy; e Department of Neurosciences, Rehabilitation, Ophthalmology, Genetics, Maternal and Child Health, University of Genoa, Genoa, Italy; f Department of Neurology, Christian Doppler Klinik, Paracelsus Medical University, Salzburg, Austria

**Keywords:** *CLN5*, Neuronal ceroid lipofuscinosis, Exome sequencing

## Abstract

**Background:**

Neuronal ceroid lipofuscinosis (NCL) is a hereditary lysosomal storage disease with progressive brain neurodegeneration. Mutations in ceroid lipofuscinosis neuronal protein 5 (CLN5) cause CLN5 disease, a severe condition characterized by seizures, visual failure, motor decline, and progressive cognitive deterioration. This study aimed to identify causative gene variants in Pakistani consanguineous families diagnosed with NCL.

**Methods:**

After a thorough clinical and neuroradiological characterization, whole exome sequencing (WES) was performed in 3 patients from 2 unrelated families. Segregation analysis was subsequently performed through Sanger sequencing

**Analysis:**

WES led to the identification of the 2 novel homozygous variants c.925_926del, (p.Leu309AlafsTer4) and c.477 T > C, (p.Cys159Arg).

**Conclusion:**

In this study, we report two novel *CLN5* cases in the Punjab region of Pakistan. Our observations will help clinicians observe and compare common and unique clinical features of NCL patients, further improving our current understanding of NCL.

## Highlights

• Neuronal ceroid lipofuscinosis is a hereditary lysosomal storage disease.
• It is characterized by progressive brain neurodegeneration.
• WES was performed in 3 patients from 2 unrelated Pakistani families.
• WES led to the identification of the 2 novel homozygous variants.
• We report a novel *CLN5* case in the Punjab region of Pakistan.

## 1 Introduction

Neuronal ceroid lipofuscinoses (NCLs) are a group of rare inherited lysosomal storage disorders leading to fatal progressive neurodegeneration [[1], [2], [3], [4]]. They affect every age and gender with a global distribution [5]. NCLs are one of the most frequent childhood-onset neurodegenerative conditions [6]. Common symptoms include seizures, progressive vision impairment, and decline in motor and cognitive functions. The diffuse involvement of the nervous system and the neurodegenerative course usually lead to premature death [7]. NCLs show a large clinical and genetic heterogeneity. To date, 13 different forms have been identified and classified according to the age of onset and affected gene [8].

Mutations in ceroid lipofuscinosis neuronal protein 5 (CLN5) cause CLN5 disease, a severe and rare form of NCL manifesting between 2 and 8 years of age, and therefore classified as late-infantile NCL (LINCL). The first symptom is usually cognitive decline with reduced learning ability often involving verbal functions. This is usually followed by seizures and visual impairment, as well as progressive decline in motor functions, usually starting with clumsiness [[9], [10], [11], [12]]. Compared to other NCLs, seizures and vision impairment occur relatively late in the disease course and seizures progression is slower than early-infantile and infantile forms [13].

Here, we identified 2 novel homozygous variants in *CLN5* in 3 patients from 2 unrelated consanguineous Pakistani families identified as part of a consortium study.

## 2 Materials and methods

### 2.1 Identification of affected individuals and collection of samples

All families were collected as part of the SYNaPS Study Group collaboration funded by The Wellcome Trust, which looks at rare disease-causing variants in consanguineous families presented with synaptopathy-related disorders. This study was approved by local institutional IRB/ethical review boards of all participating centres, and written informed consent was obtained prior to genetic testing from all the families involved. Clinical details were obtained through medical file review and clinical examination. Genomic DNA was extracted from peripheral blood samples according to standard procedures of phenol chloroform extraction. Parental blood samples and other family members were used to assess co-segregation between variants and the trait.

### 2.2 Exome sequencing

Whole exome sequencing (WES) was performed in probands as described elsewhere [14] in Macrogen, Korea. Briefly, target enrichment was performed with 2 μg genomic DNA using the SureSelectXT Human All Exon Kit version 6 (Agilent Technologies, Santa Clara, CA, USA) to generate barcoded whole-exome sequencing libraries. Libraries were sequenced on the HiSeqX platform (Illumina, San Diego, CA, USA) with 50× coverage. Quality assessment of the sequence reads was performed by generating QC statistics with FastQC (http://www.bioinformatics.bbsrc.ac.uk/projects/fastqc). Our bioinformatics filtering strategy included screening for only exonic and donor/acceptor splicing variants. In accordance with the pedigree and phenotype, priority was given to rare variants (<0.01% in public databases, including 1000 Genomes project, NHLBI Exome Variant Server, Complete Genomics 69, and Exome Aggregation Consortium [ExAC v0.2]) that were fitting a recessive (homozygous or compound heterozygous) or a de novo model and/or variants in genes previously linked to neuronal ceroid lipofuscinoses and/or other neurological disorders.

### 2.3 Sanger sequencing

Amplification reactions were performed in a total volume of 25 μl, using 50 ng of DNA, with standard FastStart PCR reagents (Roche), on an ABI Veriti Thermal Cycler (Applied Biosystems). PCR products were purified using Exo-SAP (Exonuclease I and Shrimp Alkaline Phosphatase; incubated at 37 °C for 15 min followed by inactivation by heating to 80 °C for 15 min) and sequencing PCR was performed bi-directionally using BigDye Terminator Ready Reaction Mix kit version 3.1 (Applied Biosystems) and analysed on an ABI 3730xl capillary sequencer. Electropherograms were generated on the Sequencher software to compare sequences of probands versus parents or healthy controls.

## 3 Results

### 3.1 Participants

**Fig. 1:**
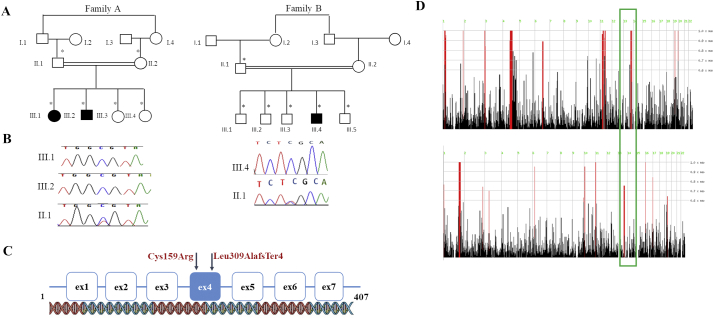
Pedigrees, Sanger sequencing, gene structure and homozygosity mapping of patients. (A) Pedigrees of the 2 families carrying the homozygous frameshift variants in. *CLN5*; c.925_926del, p.Leu309AlafsTer4 in affected individuals III.1 and III.2 and c.477 T > C, p.Cys159Arg in affected individual III.4. (B) Variants and Sanger sequencing electropherograms confirming the variants in the families. (C) The exonic organization of the *CLN5* gene depicting the location (exon 4) of the reported novel variants. (D) Homozygosity mapping analysis depicting the homozygous block (in green box) where the reported variants were identified. (For interpretation of the references to colour in this figure legend, the reader is referred to the web version of this article.)

**Fig. 2:**
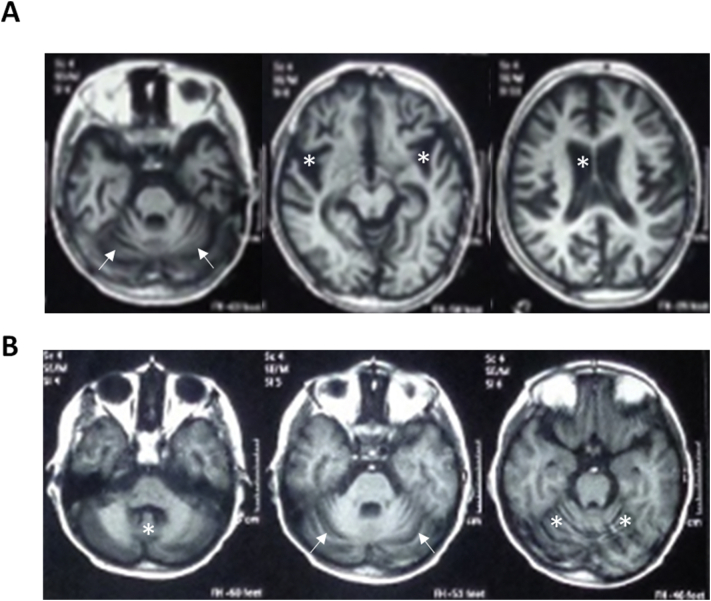
Neuroimaging findings. (A) Family A (patient 2), axial T1-weighted MRI sections show diffuse cerebral and cerebellar atrophy (white arrows), with secondary enlargement of the lateral ventricles, sylvian scissure, and subarachnoid spaces (asterisks). (B) Family B, axial T1-weighted scans showing cerebellar atrophy (white arrows) with cortical thinning and fissure enlargement of the vermis and hemispheres (asterisks).

Family A consists of four siblings, two of which are affected. They are the first- and second-born to consanguineous parents (Fig. 1A). Family history was unremarkable. Patient 1 is a 10-year-old girl and patient 2 is a 7-year-old boy. After a normal achievement of developmental milestones, both siblings experienced evident psychomotor regression. In patient 1, regression started at the age of 7 years with behavioral changes and memory loss, followed by frequent falls. She also developed visual impairment and ophthalmologic evaluation revealed bilateral optic disc pallor. She became non-ambulatory by the age of 9.5 years. The girl showed considerable cognitive decline and her speech was severely impaired, mainly consisting of incomprehensible words. Physical examination at 10 years further revealed cerebellar sings (ataxia, dysmetria, and tremors). Similarly, patient 2 started to show behavioral abnormalities (episodes of laughter, agitation, and wandering), memory loss, and impaired night vision at 6 years of age, followed by cognitive decline. His visual impairment progressed to blindness and fundoscopic examination revealed bilateral optic disc pallor. In the following months, he experienced frequent falls and became unable to walk, even if supported. At 7 years, he was only able to crawl and sit unsupported. His speech was limited to babbling and neurological examination revealed ataxia and tremors. Both siblings suffered from recurrent and refractory myoclonic seizures, starting at the age of 7.5 years and 6 years for patient 1 and 2, respectively. EEG showed multifocal epileptiform discharges. In patient 1, brain MRI revealed diffuse cerebellar and cerebral atrophy. Neuroimaging abnormalities in patient 2 consisted instead of enlarged ventricles and subarachnoid spaces, cerebellar atrophy with prominent folia, and white matter hyperintensities with predominant involvement of the posterior limb of the internal capsules (Fig. 2A). Patient 1 died at the age of 11 years. Her brother is currently alive and presents refractory epilepsy, severe cognitive deterioration, and worsening ataxia.

Family B relates to a 10-year-old boy born from consanguineous parents (Fig. 1A) with deterioration of previously acquired skills following first seizure at the age of 6 years. Before occurrence of this first symptom, development was normal and milestones had been reached age-appropriately. First manifestation were myoclonic jerks followed by drop attacks leading to frequent falls during the first year after disease onset. At 7 years of age, he additionally developed focal impaired awareness motor seizures, which progressed to bilateral tonic-clonic seizure at the age of 9 years. Seizures are resistant to drug treatment (currently partial response to valproic acid, previous medication with levetiracetam, and clonazepam failed to control or reduce seizures). In parallel with seizure progression a regression of motor, verbal, and memory functions was observed. Motor regression started as a slight imbalance of gait and progressed to complete inability to walk for the last 2 years. However, the patient was still able to stand and sit without support. Verbal functions deteriorated completely (language limited to 1 to 2 words) as well as memory functions. Over the last year, the family also noticed visual impairment, with the inability to fix objects or faces. Neurological examination at a young age, showed excessive drooling, axial ataxia with positive cerebellar signs (dysmetria, incoordination, no nystagmus), decreased muscle tone, normal reflexes, and up going planters. The EEG showed multifocal epileptiform discharges and brain MRI revealed cerebellar atrophy (Fig. 2B).

### 3.2 Genetic analysis

Two novel homozygous variants in *CLN5* were identified in the studied families (Fig. 1B). In family A, the novel homozygous missense variant ENST00000377453.8: c.477 T > C, p.Cys159Arg (chr13:76,996,037) was identified in *CLN5*. This variant was found within the most significant homozygous block (chr13: 73,075,970-77,070,727) in exon 4 (Fig. 1D top panel). Both parents and siblings were found to be heterozygous carriers. In family B, we found the novel *CLN5* frameshift variant ENST00000377453.8: c.925_926del, p.Leu309AlafsTer4 (chr13:77,000,816). This deletion of two nucleotides at c.925_926 in exon 4 is predicted to cause a frameshift. The variant is present within a significant homozygosity block (chr13: 69,719,583-77,217,964) (Fig. 1D bottom panel). Both parents were found to be carriers. *In-silico* analysis by SIFT and Polyphen tools confirmed the severe impact of these *CLN5* variants as damaging amino acid substitutions, most probably resulting in a loss of function effect.

## 4 Discussion

NCLs are classified into four major subtypes which include infantile (INCL), later infantile (LINCL), juvenile (JNCL) and adult (ANCL) [15]. The onset of loss of vision progressing to blindness, regression in cognitive and motor abilities, and epileptic abnormalities in early teen-age might underly the change into NCLs. The onset of such symptoms varies in accordance to the type of NCL in every individual [16]. CLN5 disease was originally identified as a rare variant of NCLs restricted to Finnish and other Northern European populations [17]. Since then, more cases from Italy and China have been identified and in vitro functional expression studies in HEK293 cells showed retention of lysosomal CLN5 in the endoplasmic reticulum (not reaching the lysosome).

CLN5 is a soluble protein present in the lysosomal lumen, with important roles such as neurogenesis and neuronal repair, synaptic endocytosis and autophagy [[18], [19], [20], [21], [22]]. It is encoded by the *CLN5* gene (OMIM: 608102) and plays a role in the retrograde trafficking of lysosomal sorting receptors SORT1 and IGF2R from the endosomes to the trans-Golgi network [21]. NCL proteins localize either to lysosomes (CLN1, CLN2, CLN3, CLN5, CLN7, CLN10, CLN12 and CLN13), the endoplasmic reticulum (CLN6 and CLN8), or the cytosol linked to vesicular membranes (CLN4 and CLN14) [23]. Despite all of them affecting lysosomal degradation process, the NCL-associated proteins display high heterogeneity in cellular localization. Recent evidence has showed that CLN5 may also localize extracellularly; however very little is known about its function outside the cell [24,25].

In the two Pakistani families, we identified two novel homozygous variants in *CLN5* implicated in NCLs, the c.477 T > C, p.Cys159Arg and the c.925_926del, p.Leu309AlafsTer4. Our subjects are also the second *CLN5* cases in the Pakistani population, after the first Asian sibship from Pakistan was reported back in 2009 [26]. To date, most cases of *CLN5* disease have been described in Finnish and norther European countries as well as in Italy and China. Our findings indicate that *CLN5* pathogenic variants are also present in the South Asian-Punjab area, resulting in a more worldwide distribution of CLN5 disease. The clinical presentation of these patients along with the MRI findings of cerebellar atrophy, further implicate neurodegeneration in the pathogenesis of the CLN5 disease, as indicated in previous studies.

Our report highlights a possible founder effect for CLN5 in South Asia and broaden the phenotypic spectrum of this condition. Next generation sequencing based studies involving populations from different ethnic backgrounds will help to understand the exact worldwide distribution of the rarest NCLs in the future, also contributing to the further delineation of the associated clinical phenotypes. In the long-term, this information will help affected families in terms of clinical management and prognosis, as well as family planning and prenatal diagnosis.
